# Sodium‐glucose cotransporter type 2 channel inhibitor: Breakthrough in the treatment of neutropenia in patients with glycogen storage disease type 1b?

**DOI:** 10.1002/jmd2.12278

**Published:** 2022-03-02

**Authors:** Magdalena Kaczor, Milena Greczan, Karolina Kierus, Ewa Ehmke vel Emczyńska‐Seliga, Elżbieta Ciara, Barbara Piątosa, Dariusz Rokicki, Janusz Książyk, Dorota Wesół‐Kucharska

**Affiliations:** ^1^ Department of Paediatrics, Nutrition and Metabolic Diseases Children's Memorial Health Institute Warsaw Poland; ^2^ Department of Paediatrics, Rheumatology, Immunology and Metabolic Bone Diseases Medical University of Bialystok Białystok Poland; ^3^ Department of Medical Genetics Children's Memorial Health Institute Warsaw Poland; ^4^ Histocompatibility Laboratory Children's Memorial Health Institute Warsaw Poland

**Keywords:** empagliflozin, G‐CSF, GSD 1b, neutropenia, SGLT2 inhibitor

## Abstract

Glycogen storage disease type 1b (GSD 1b) is an inherited metabolic defect caused by biallelic mutations in the *SLC37A4* gene encoding microsomal glucose‐6‐phosphate (G6P) transporter in the endoplasmic reticulum (ER) membrane. Ineffective G6P transport into the ER leads to hypoglycaemia, hyperlactatemia, hyperuricemia, hypertriglyceridemia, hepato‐ and/or nephromegaly. Clinical manifestations of the disease include recurrent, severe infections and inflammatory bowel (Crohn‐like) caused by neutropenia and diminished bactericidal and fungicidal activity of neutrophils. Granulocyte colony‐stimulating factor (G‐CSF) administration is currently a standard therapy to prevent adverse effects of neutropenia, but the treatment is associated with a high risk of severe side effects. On the other hand, short‐treatment with sodium‐glucose cotransporter type 2 inhibitor – empagliflozin (EMPA) was reported to act directly on the mechanism of neutropenia and neutrophil dysfunction in GSD 1b. We observed significant improvement in clinical and laboratory parameters after introducing EMPA to treatment, that is reduced frequency of infections, lower number of bowel movements, and improved postoperative wound healing. EMPA is effective in the treatment of neutropenia in our GSD 1b patients, which allows for dose reduction and even withdrawal of G‐CSF. We did not observe any significant side effects of EMPA treatment in our patients.

**Synopsis**

Glycogen storage disease type 1b (GSD 1b) is an inherited metabolic defect caused by balletic mutations in the *SLC37A4* gene encoding microsomal glucose‐6‐phosphate transporter in the endoplasmic reticulum membrane. The main symptoms of the disease include short stature, hepatomegaly, hypoglycaemia, hyperlactatemia, hypertriglyceridemia, and neutropenia.

Neutropenia leads to severe infections and the development of inflammatory bowel disease despite G‐CSF treatment.

We observed significant improvement in clinical and laboratory parameters after introducing empagliflozin to treatment without significant side effects.

## 1 INTRODUCTION

Glycogen storage disease type 1b (GSD 1b) is a rare (prevalence 1: 500 000) autosomal recessive metabolic defect caused by biallelic mutations in the *SLC37A4* gene. It leads to a deficiency of microsomal glucose‐6‐phosphate (G6P) transporter in the endoplasmic reticulum (ER) membrane. G6P hydrolysis to glucose is possible only inside ER. Therefore transporter absence/deficiency in ER results in hypoglycaemia.^1^, ^2^ An increased G6P in cytoplasm results in excessive stimulation of metabolic processes, leading to laboratory abnormalities typical for GSD 1b (hyperlactatemia, hypertriglyceridemia, hyperuricemia, and hypercholesterolemia).^1^, ^2^, ^3^ In addition, poorly metabolised glycogen and fats accumulate in the liver and kidneys, leading to their enlargement and dysfunction. Hepatic adenomas, abnormal proximal or distal renal tubule function, and progressive glomerular fibrosis were also observed.^1^

Metabolic aberrancies in G6P transport also negatively affect neutrophil count and function. As a result, GSD 1b patients demonstrate an increased frequency of severe infections and predisposition to inflammatory bowel (Crohn‐like) disease.^1^, ^2^, ^3^ Premature apoptosis and reduced bactericidal and fungicidal activity of neutrophils in GSD 1b patients are caused by an excessive accumulation of 1,5 anhydroglucitol‐6‐phosphate (1,5 AG6P) in cell cytoplasm.^2^, ^3^ 1,5 AG6P inhibits hexokinase and negatively affects the conversion of glucose into G6P, thereby reducing the amount of substrate for production of energy and free oxygen radicals.^2^, ^3^

The granulocyte colony‐stimulating factor (G‐CSF) treatment of neutropenia in patients with GSD 1b aims to improve the absolute neutrophil count and reduce the severity of infections. Unfortunately, pathological effects are not eliminated, and life‐threatening infections are still observed, while inflammatory bowel disease is often poorly controlled.^3^, ^4^ In addition, the ineffective elimination of toxic metabolites from neutrophils reduces G‐CSF efficacy in GSD1b.^2^

Empagliflozin (EMPA), a renal sodium‐glucose cotransporter type 2 (SGLT2) inhibitor approved for the treatment of diabetes mellitus type 2, inhibits the reuptake of 1,5 anhydroglucitol (1,5 AG) in renal tubules.^3^, ^5^ Lowering the serum concentration of 1,5 AG respectively reduces the concentration of 1,5 AG6P in the neutrophil cytoplasm. What in patients with GSD 1b leads to the unblocking of glycolysis and pentose phosphate cycle, thus prolonging the survival of the neutrophil and improving its function.^3^, ^5^

## 2 AIM

The aim of this study is to describe the effects of EMPA treatment (3–18 months of treatment) in four patients with GSD 1b respect to clinical symptoms and laboratory parameters.

## 3 MATERIAL AND METHODS

The study included four patients with GSD 1b, two girls and two boys, remaining under the combined care of the Department of Paediatrics, Nutrition and Metabolic Diseases, Children's Memorial Health Institute (IP‐CZD) in Warsaw and the Department of Paediatrics, Rheumatology, Immunology and Metabolic Bone Diseases, Medical University of Bialystok. Due to unsatisfactory improvement on G‐CSF, we started treatment with EMPA. Clinical data and laboratory parameters in similar follow‐up periods before and after the introduction of EMPA were retrospectively analysed to evaluate the effects of EMPA treatment.

The collected data included the number of urgent hospitalisations due to infection, the number of infections requiring antibiotic therapy, the mean number of stools per day, and the average number of days per month with mucosal lesion before and after the implementation of EMPA therapy. In addition, we evaluated the metabolic response by assessing the concentration of glucose, lactic acid, uric acid, and triglyceride in serum and by evaluating the absolute neutrophil count. Finally, results of all analysed parameters were compared for similar periods before and after introducing EMPA treatment.

## 4 CASES DESCRIPTION

P1: Male patient, currently 17.5 years old, was born by Caesarean section from pregnancy G1 at 41 weeks, with Apgar score of 10 and birth weight of 3.800 g. Congenital pneumonia complicated the neonatal period. At 2 months of age, perirectal abscess drainage was required. Two weeks later, the patient developed a generalised infection of unknown aetiology. Hepatomegaly, hypoglycaemia of 1.7 mmol/L after a 3‐h feeding break, and neutropenia (200 cells/μl, N > 1000) were detected. Molecular testing revealed a homozygous *SLC37A4* variant, c.1042_1043del.

The boy was repeatedly hospitalised during several subsequent months due to hypoglycaemia, recurrent severe bacterial infections (pneumonia, bilateral otitis), episodes of stomatitis, and/or diarrhoea. Due to severe, persistent neutropenia (100–200 cells/μl, N > 1000), G‐CSF at 5 μg/kg/24 h dose was introduced to the therapy when the patient was 22 months old. By 16 years of age, the boy was hospitalised approximately 50 times due to recurrent bacterial infections, diarrhoea, mouth sores, and skin abscesses. Despite G‐CSF treatment, no significant increase in the neutrophil count was achieved. At 3 years, the patient developed severe Crohn‐like bowel disease and periodically required parenteral nutrition. At 16 years of age, the patient started zoledronic treatment due to significant thinning of bone structure and numerous vertebral compression fractures found in dual‐photon X‐ray absorptiometry (DXA).

Despite G‐CSF and mesalazine, during the year preceding the introduction of EMPA, the patient demonstrated poor control of inflammatory bowel disease, and he experienced several episodes of severe infections (Table 1). Laboratory tests revealed severe neutropenia but a normal concentration of lactic acid, uric acid and triglycerides in serum (Table 1). Due to unsatisfactory response to G‐CSF (2.7 μg/kg/24 h), a treatment with EMPA was initiated (of label use).

**TABLE 1:** Clinical course and results of laboratory tests in patients with glycogen storage disease type 1b (GSD 1b) without versus with empagliflozin (EMPA)

Patient/current age	P1/17 years		P2/13 years		P3/9 years		P4/17 months	
Without/with EMPA	Without EMPA 1.5 years before EMPA	EMPA ‐ Final dose: 0.3 mg/kg/24 h ‐ Treatment duration: 1.5 years	Without EMPA 1 year before EMPA	EMPA ‐ Final dose 0.4 mg/kg/24 h ‐ Treatment duration: 1 year	Without EMPA 1 year before EMPA	EMPA ‐ Final dose 0.4 mg/kg/24 h ‐ Treatment duration: 1 year	Without EMPA 6 months before EMPA	EMPA ‐ Final dose: 0.4 mg/kg/24 h ‐ Treatment duration: 6 months
Number of urgent hospitalisations due to infection	9	0	1	0	6	3	4	0
Number of infections requiring antibiotic therapy	3	1	5	1	5	2	0	0
Mean number of stools/24 h	8–10	3–5	7–8	3–4	6–8	2–3	1–2	1–2
Average number of days per month with mucosal lesions	10–12	0 (from 1 year)	10	0 (from 6 months)	6	2	0	0
Neutrophil count (cell/μl)^a^	M: 131 R: 74–176	M: 440 R: 320–572	M: 470 R: 190–790	M: 1040 R: 550–1210	M: 625 R: 240–730	M: 815 R: 650–860	M: 890 R: 860–950	M: 950 R: 500–3000
Mean G‐CSF dose (μg/kg/24 h)	2.7	0 (from 6 months)	2.5	0 (from 6 months)	6	3	2	0 (from 3 months)
Lactate (mmol/L, N < 2.22)^a^	M: 1.06 R: 1.03–1.09	M: 1.22 R: 1.14–1.30	M: 2.94 R: 1.11–5.88	M: 2.48 R: 1.93–3.28	M: 1.39 R: 1.05–2.15	M: 1.23 R: 1.04–1.4	M: 3.77 R: 2.66–5.66	M: 5.55 R: 5.49–6.1
Uric acid (μmol/L, N < 416)^a^	M: 371.7 R: 303–475.8	M: 280.75 R: 243.87–327.14	M: 713.8^b^ R: 374.72–933.84	M: 386.62^c^ R: 333.1–422.31	M: 401.5 R: 291.5–499	M: 237.92 R: 196.3–261.7	M: 410.4 R: 83.3–434	M: 136.8 R: 107–166.54
Triglycerides (mmol/L, N < 1.71)^a^	M: 0.71 R: 0.6–0.75	M: 0.68 R: 0.63–0.74	M: 8.74 R: 7.49–1.99	M: 4.69^d^ R: 3.37–14.31	M: 1.15 R: 0.81–1.93	M: 1.19 R: 1.0–1.31	M: 7.34 R: 2.32–12.11	M: 7.92 R: 7.91–7.92

Abbreviations: G‐CSF, granulocyte colony‐stimulating factor; M, median; N, normal; R, range.

^a^ Results of laboratory tests in patients without infection.

^b^ Patient additionally treated with allopurinol.

^c^ Patient additionally treated with febuxostat.

^d^ Patient additionally treated with fenofibrate.

P2: Male patient, currently 13 years old, was born from normal gestation and labour. A single hypoglycaemia episode occurred on the 8th day of life. During the diagnostic process of recurrent infections (4× acute otitis media, 2× upper airway infection, purulent inflammation of the axillary pit, and soft tissue infection in the eye region) performed at 9 months of age, lipid disorders, hypoglycaemia, hepatomegaly, and neutropenia were discovered. The patient was transferred to the Department of Metabolic Diseases in Warsaw. At 9 months of life, the patient demonstrated normal body weight (10 kg, 50th pc) and length (69 cm, 25th pc). Laboratory tests revealed hyperlactatemia (4.6 mmol/L, N < 2.22), hypertriglyceridemia (15.06 mmol/L, N < 1.71), hyperaminotransferasaemia (AST/ALT 108/149 IU/L, N < 40/30), neutropenia (115 cells/μl, N > 1000 cells/μl), and high normal uric acid (410 μmol/L, N < 416) (Table 1). Abdominal ultrasound revealed a normal renal image but confirmed significant hepatomegaly with no abnormalities in the echo pattern. GSD 1b was suspected and confirmed at 9 months of age, with compound heterozygosity for the variants c.1042_1043 del CT and c.341A>G revealed in *SLC37A4* gene (Table 2).

**TABLE 2:** Patients with glycogen storage disease type 1b (GSD 1b) summary

	P1	P2	P3	P4
Molecular analysis	c.1042_1043del, (p.Leu348Valfs*53) homozygous	c.[1042_1043del]; [341A>G], (p.[Leu348Valfs*53]; [Gln114Arg])	c.1042_1043del, (p.Leu348Valfs*53) homozygous	c.[1042_1043del]; [1175del], p.[Leu348Valfs*53]; [Ser392Ilefs*11]
Initial presentation	INF, HMG, HGL	HGL, INF, HMG, HTG, HLA	HGL, INF, HTG, HLA	INF, HLA, HTG
Age at diagnosis (months)	4	9	1	2.5
Gender	Male	Male	Female	Female
Age/dose when starting G‐CSF treatment	2 years old 5 μg/kg/24 h	2 years old 2.5 μg/kg/24 h	15 months 5 μg/kg/24 h	8 months 4 μg/kg/24 h
Respiratory burst before/after starting G‐CSF treatment	Reduced/reduced	Reduced/normal	Reduced/normal	Reduced/normal
Total follow‐up period (years)	17	13	8	1
IBD, age (years) start of mesalazine treatment	3	9	2.5	**‐**
Hypertension	No	Yes	No	No

Abbreviations: G‐CSF, granulocyte–macrophage colony‐stimulating factor; HGL, hypoglycemia; HLA, high lactic acid; HMG, hepatomegaly; HTG, hypertriglyceridemia; IBD, inflammatory bowel disease; INF, severe/recurrent infection.

Due to frequent infections (3× upper airway infections, 2× skin abscesses per year) and low neutrophil count (900 cells/μl), G‐CSF was introduced at a 2.5 μg/kg/24 h when the patient was 2 years old. Despite normalisation of the neutrophil count, the patient went through numerous infections with three episodes of acute otitis media, four episodes of pneumonia and several surgeries for granulomatous otitis media and renal abscesses during 10 years of follow‐up under treatment with G‐CSF. Additionally, the patient developed inflammatory bowel disease requiring mesalazine at 9 years (initially 2× 500 mg, increased to 4× 500 mg due to unsatisfactory results).

We started an “off‐label” EMPA treatment because of recurrent infections, low neutrophil count, and lack of inflammatory bowel disease remission on G‐CSF (2.5 μg/kg/24 h) and mesalazine (40 mg/kg/day).

During 1 year preceding the introduction of EMPA, the patient experienced submandibular lymphadenitis, three episodes of respiratory tract infection, and one episode of skin abscess. In addition, multiple stools per day (on average 7–8) and ulcer‐like lesions in the mouth for 10 days per month were observed (Table 1). Laboratory tests performed before the introduction of EMPA showed normal lactic acid concentration in serum, but persistent neutropenia, high concentration of uric acid despite the use of allopurinol, and elevated levels of triglycerides (Table 1). Nutritional treatment did not affect hypertriglyceridemia and hyperuricemia. Fenofibrate and febuxostat were added before initiation of the EMPA therapy.

P3: Female patient, currently 9 years old, with uneventful gestational and neonatal history, experienced an episode of hypoglycaemia accompanying bilateral otitis media of *Streptococcus agalactiae* aetiology on 2nd day of life. Surgery on the 20th day of life was required to remove a skin abscess in the paranasal infraorbital region with subsequent generalised infection by *Staphylococcus aureus* (*MSSA*). At 5 months of age, an in‐depth diagnostic procedure was initiated after an episode of generalised seizures accompanying low serum glucose (1.66 mmol/L). Hepatomegaly and neutropenia (190 cells/μl) (Table 1) suggested that the patient may suffer from GSD 1b. Mutation analysis of the *SLC37A4* gene revealed a homozygous variant (c. 1042_1043del) confirming the diagnosis (Table 2). At 15 months of age, recurrent infections, chronic neutropenia, and poor NADPH oxidase activity identified as abnormal “respiratory burst” (Table 2) prompted to use G‐CSF at a dose of 5 μg/kg/24 h. Gastrostomy was performed at age of 18 months due to feeding difficulties and was complicated by delayed wound healing. The frequency of infections decreased after the introduction of G‐CSF. However, the patient developed colitis at 2.5 years of age. Despite mesalazine treatment initiated for persistent diarrhoea, pathologic lesions were found by colonoscopy (Table 2). The patient was repeatedly hospitalised, most frequently due to purulent lesions in the gastrostomy area requiring surgical treatment. The gastrostomy tube could only be removed at the age of eight due to long‐lasting feeding difficulties. Recurrent sinusitis, otitis, and poor control of inflammatory bowel disease despite treatment with G‐CSF and mesalazine prompted introduction of an EMPA (off label use). The dose was gradually increased to a final dose of 0.4 mg/kg/24 h.

On daily administration of G‐CSF (6 μg/kg/24 h) and mesalazine (2× 500 mg), the parameters reflecting metabolic control (lactic acid, uric acid, and triglycerides concentration in serum) remained within normal limits. However, during the year preceding the introduction of EMPA, a gradual reduction of neutrophil count has been observed. The patient has been hospitalised six times for severe infections, and five times she required outpatient therapy with antibiotics (Table 1). Remission of inflammatory bowel disease has not been achieved (Table 1).

**FIGURE 1:**
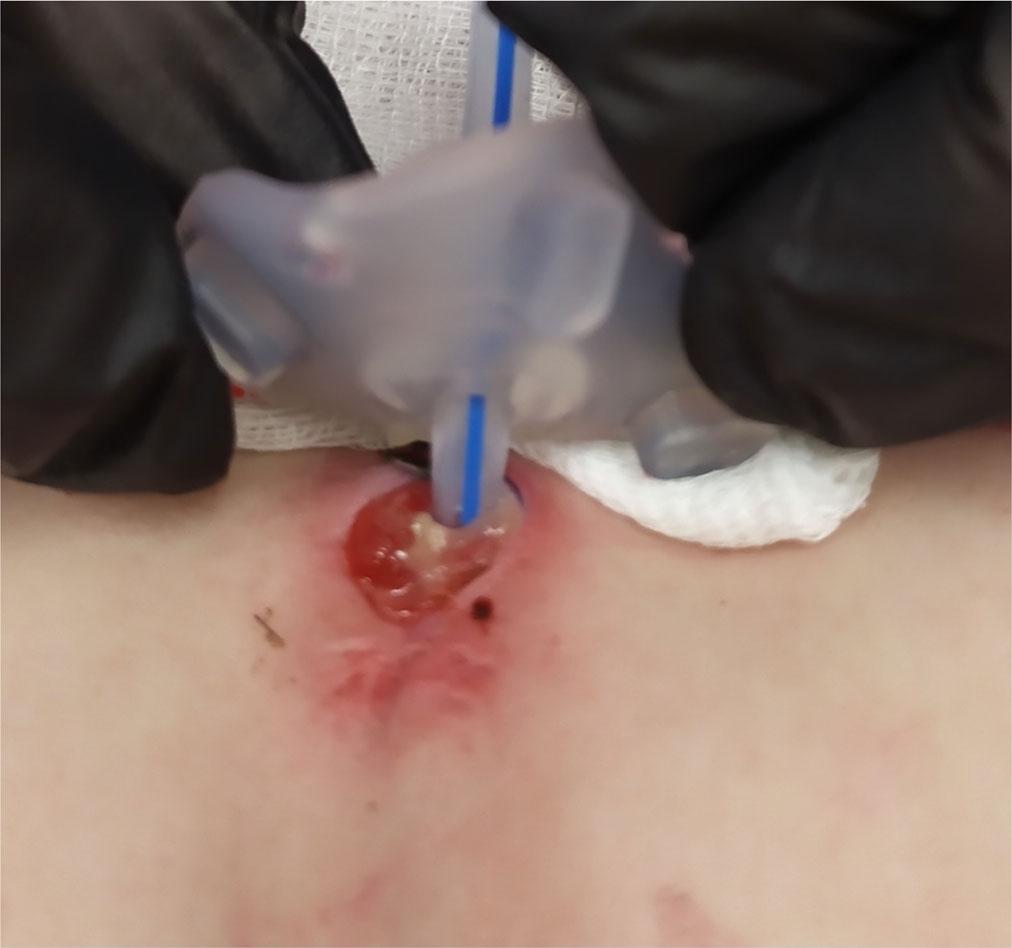
Postoperative wound healing before EMPA treatment (the gastrostomy area 26 days after surgery)

P4: Female patient, currently 17 months old, with uneventful neonatal history, developed a generalised infection of *MSSA* aetiology at 2 months of age. Laboratory results demonstrated a high triglyceride level (18–27.36 mmol/L) (Table 2). On admission to the Department of Metabolic Diseases in the Children's Memorial Health Institute at 2.5 months of age, the girl weighed 5 kg (3rd–10th c), and her length was 55 cm (50th c). Laboratory tests revealed hyperlactatemia (13.98 mmol/L, N < 2.22), hypertransaminasemia (ALT/AST 92/29 IU/L, N < 30/40), and hypertriglyceridemia (20.62 mmol/L, N < 1.71). Hepatomegaly, without nephromegaly or abnormal echo pattern, was found in ultrasound examination (Table 2). Initially, no significant neutropenia was observed (780–890 cells/μl), but NADPH oxidase activity was reduced. GSD 1b was confirmed by identification of biallelic mutations in *SLC37A4* gene (c.1042_1043del and c.1175del) (Table 2). We modified the diet and started prophylactic amoxicillin for neutropenia. At 6 months of age, the patient required hospitalisation due to generalised infection with *Enterococcus faecalis*. At 8 months of age, G‐CSF was introduced at a dose of 4 μg/kg/24 h due to recurrent generalised infections and persisting neutropenia. The normalisation of neutrophil count and NADPH oxidase activity has been observed after introduced G‐CSF (Table 2). Two months later, increasing difficulties in taking regular oral meals necessitated the emergence of a gastrostomy. The procedure was complicated by delayed wound healing. Once again, the neutrophil count and NADPH oxidase activity were checked and proved to be in normal range. Due to the exhaustion of all available methods of treatment (local and generalised antibiotic, disinfectants etc.) taking into consideration promising reports on the beneficial effects of EMPA on wound healing in patients with GSD 1b, on day 26 after the surgery, EMPA (off label use) was introduced (Figure 1).

## 5 RESULTS

P1: For 1 year and 6 months, EMPA (0.3 mg/kg/24 h) was administered in combined treatment with G‐CSF (2.7 μg/kg/24 h). Good general condition, improvement in blood differential, and lack of infections prompted reducing the G‐CSF dose to 1.4 μg/kg/24 h and subsequently withdrawing it. Currently, the boy is off G‐CSF for 6 months (Table 1). Following the introduction of EMPA, a significant reduction in the number of infectious episodes and the number of stools per day with the resolution of mouth sores or skin abscesses (Table 1). The patient experienced a single episode of pulmonary infection requiring antibiotic therapy in an outpatient setting after the introduction of EMPA (Table 1). Metabolic control and neutrophil counts remain within normal limits (Table 1). Despite the expected glucosuria, no hypoglycaemia or urinary tract infections were observed during treatment.

P2: We started filgrastim (2.5 μg/kg/24 h) and EMPA (0.4 mg/kg/24 h), and for 1 year, the patient received both. As a result, we observed a reduced frequency of infections, mean number of stools per day, and lower incidence of mouth sores. Significant clinical improvement and satisfactory neutrophil counts (above 1000 cells/μl) allowed gradual withdrawal of G‐CSF (Table 1). Currently, the patient is off G‐CSF for 6 months, with a single upper respiratory tract infection treated symptomatically. The number of stools was significantly reduced, and no mucosal lesions were observed (Table 1). Metabolic control, that is serum concentration of lactic acid, uric acid and triglycerides, improved, although these might be caused by simultaneous introduction of fenofibrate and febuxostat. No adverse reactions to EMPA treatment were observed.

P3: Combined treatment with G‐CSF (6 μg/kg/24 h) and EMPA (0.4 mg/kg/24 h) resulted in clinical improvement, that is reduced frequency of hospitalisations for infections down to three per year, the need for outpatient treatment down to two per year, with fewer stools per day and only a few mouth sores (Table 1). We recorded no urinary tract infections and a single episode of hypoglycaemia (1.44 mmol/L) related to the delayed supply of the night meal. Due to persistently low neutrophil count (median number 815 cells/μl [range: 650–860 cells/μl]), G‐CSF after a year of combined treatment with EMPA was not withdrawn, but its dose was reduced by 50% (Table 1).

**FIGURE 2:**
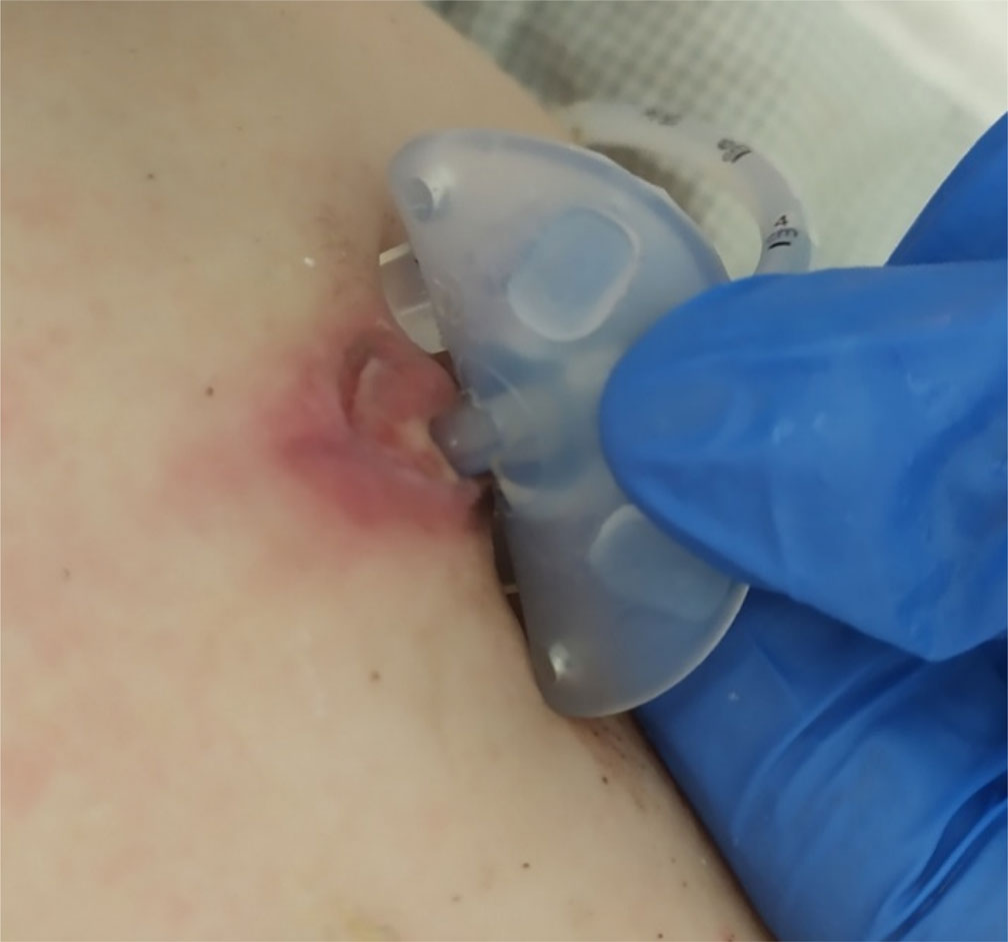
Postoperative wound healing 3 days after introduction of EMPA treatment (the gastrostomy area 29 days after surgery)

P4: The patient was treated with G‐CSF (2 μg/kg/24 h) and EMPA (0.4 mg/kg/dl) for 2 months. Acceleration of wound healing was observed 3 days after introducing EMPA (Figure 2), while 2 days later, it was possible to discharge the patient home. Although EMPA was used for a brief period (6 months), an increase in the neutrophil count was observed (Table 1). Two months after the introduction of EMPA, it was possible to withdraw G‐CSF (Table 1). No infections occurred, and no adverse reactions to treatment were observed.

## 6 DISCUSSION

In the maturation process, neutrophils lose most of their mitochondria and become dependent on the energy coming from anaerobic glycolysis. As a result, glucose 6‐phosphate, the product of the first glycolysis stage, is also used in the pentose phosphate cycle. The pentose phosphate pathway serves, among others, in production of NADPH, which is used by NADPH oxidase to produce free oxygen radicals.^2^

1,5 AG is common in food, but its function in vivo is still unknown.^2^, ^3^ Under physiologic conditions, 1,5 AG6P is transported to the ER, converted by glucose‐6‐phosphatase 3 (G6PC3) to 1,5 AG and phosphate, and eliminated from the cell.^2^, ^3^ In patients with GSD 1b, the transporter deficiency causes 1,5 AG6P accumulation in the cytoplasm of neutrophils. Excess of 1,5 AG6P inhibits hexokinase and the conversion of glucose into glucose‐6‐phosphate and therefore insufficient cell energy production. This results in energy deficiency and impaired production of free oxygen species.^2^, ^3^

Treatment with G‐CSF positively affects the neutrophil count, it reduces frequency and severity of infections observed in patients with GSD 1b. Unfortunately, pathological effects are not eliminated and life‐threatening infections are still observed, while inflammatory bowel disease is often poorly controlled. Moreover, such treatment is not free of adverse reactions, with osteoarticular pain, gingival hypertrophy and bleeding, splenic enlargement often accompanied by hypersplenism, and an increased risk of hematologic neoplasms.^4^, ^6^

EMPA inhibits the reabsorption of glucose from primary urine, therefore leading to reduced glucose concentration in serum. The drug is successfully used in adult patients to control diabetes mellitus type 2, providing cardio‐ and nephroprotective effects due to reduced blood pressure.^7^, ^8^ EMPA has also been reported to inhibit the reuptake of 1,5 AG in renal tubules.^3^, ^8^ Lowering the serum concentration of 1,5 AG reduces the concentration of 1,5 AG6P in the neutrophil cytoplasm. This decrease leads to an unblocking of the glycolysis and the pentose phosphate cycle, thus extension in the survival and the improvement in function of neutrophils^3^, ^5^ .

The most common adverse reactions of EMPA treatment include episodes of hypoglycaemia (when the drug is used with another hypoglycaemic agent) and an increased frequency of genitourinary infections.^3^, ^6^, ^7^, ^8^ None of these has was observed in our patients. The number of neutrophils increased, and the frequency of infections was reduced.^3^, ^8^

Auspicious results of treating neutropenia with EMPA in patients with GSD 1b suggest a need to perform further studies. In addition, considering the reported nephro‐ and cardioprotective effect of the drug, a longer follow‐up might show whether there are any other beneficial effects of EMPA treatment in patients with GSD 1b.

## 7 CONCLUSIONS

The case reports presented above confirm the efficacy of EMPA in the short‐term treatment of neutropenia in GSD1b patients, as described by Wortmann et al. and Grünert et al.^3^, ^5^ EMPA treatment results in significant clinical improvement. Through the therapy, we observed: the reduced frequency of infections, the decrease in number of bowel movements and significant improvement in wound healing (Table 1). The EMPA therapy led to partial or a complete withdrawal of G‐CSF in all patients. Clinical improvement seems to be related mainly to amelioration of neutrophil function. None of the patients revealed significant adverse reactions to EMPA treatment. In all four patients, the EMPA therapy is continued.

## CONFLICT OF INTEREST

Magdalena Kaczor, Milena Greczan, Karolina Kierus, Ewa Ehmke vel Emczyńska‐Seliga, Elżbieta Ciara, Barbara Piątosa, Dariusz Rokicki, Janusz Książyk and Dorota Wesół‐Kucharska declare that they have no conflict of interest.

## AUTHOR CONTRIBUTIONS

Magdalena Kaczor: designed the study, collected data, data analyses, data interpretation and wrote the manuscript. Milena Greczan: designed the study, collected data, data analyses, data interpretation, contributed to the drafting of the manuscript. Karolina Kierus: designed the study, collected data, data analyses, data interpretation, contributed to the drafting of the manuscript. Ewa Ehmke vel Emczyńska‐Seliga: collected data, contributed to the drafting of the manuscript, critically reviewed the manuscript. Elżbieta Ciara: collected data, contributed to the drafting of the manuscript, critically reviewed the manuscript. Barbara Piątosa: collected data, data analyses, contributed to the drafting of the manuscript, critically reviewed the manuscript. Dariusz Rokicki: designed the study, data analyses, data interpretation, contributed to the drafting of the manuscript, critically reviewed the manuscript. Janusz Książyk: data analyses, contributed to the drafting of the manuscript, critically reviewed the manuscript. Dorota Wesół‐Kucharska: corresponding author, designed the study, collected data, data analyses, data interpretation, wrote part of the manuscript, critically reviewed the manuscript.

## INFORMED CONSENT

All procedures followed were in accordance with ethical standards of the responsible committee on human experimentation (institutional and national) and with the Helsinki Declaration of 1975, as revised in 2000. Informed consent was obtained from all patients for being included in the study.

## ANIMAL RIGHTS

This article does not contain any studies with animal subjects performed by any of the authors.

## Data Availability

Data archiving is not mandated but data will be made available on reasonable request.
